# Supplementary material for: Activation of interferon regulatory factor 3 by replication-competent vaccinia viruses improves antitumor efficacy mediated by T cell responses
Source: Mol Ther Oncolytics. 2021 Jun 4;22:399–409. doi: 10.1016/j.omto.2021.06.001 (PMC8430050; doi:10.1016/j.omto.2021.06.001)
Supplement: Document S1. Figures S1–S4 [file mmc1.pdf]

OMTO, Volume *22*

Supplemental information

Activation of interferon regulatory factor 3 by replication-competent vaccinia viruses improves antitumor efficacy mediated by T cell responses

Stephanie Riederer, Robert Fux, Michael H. Lehmann, Asisa Volz, Gerd Sutter, and Juan J. Rojas

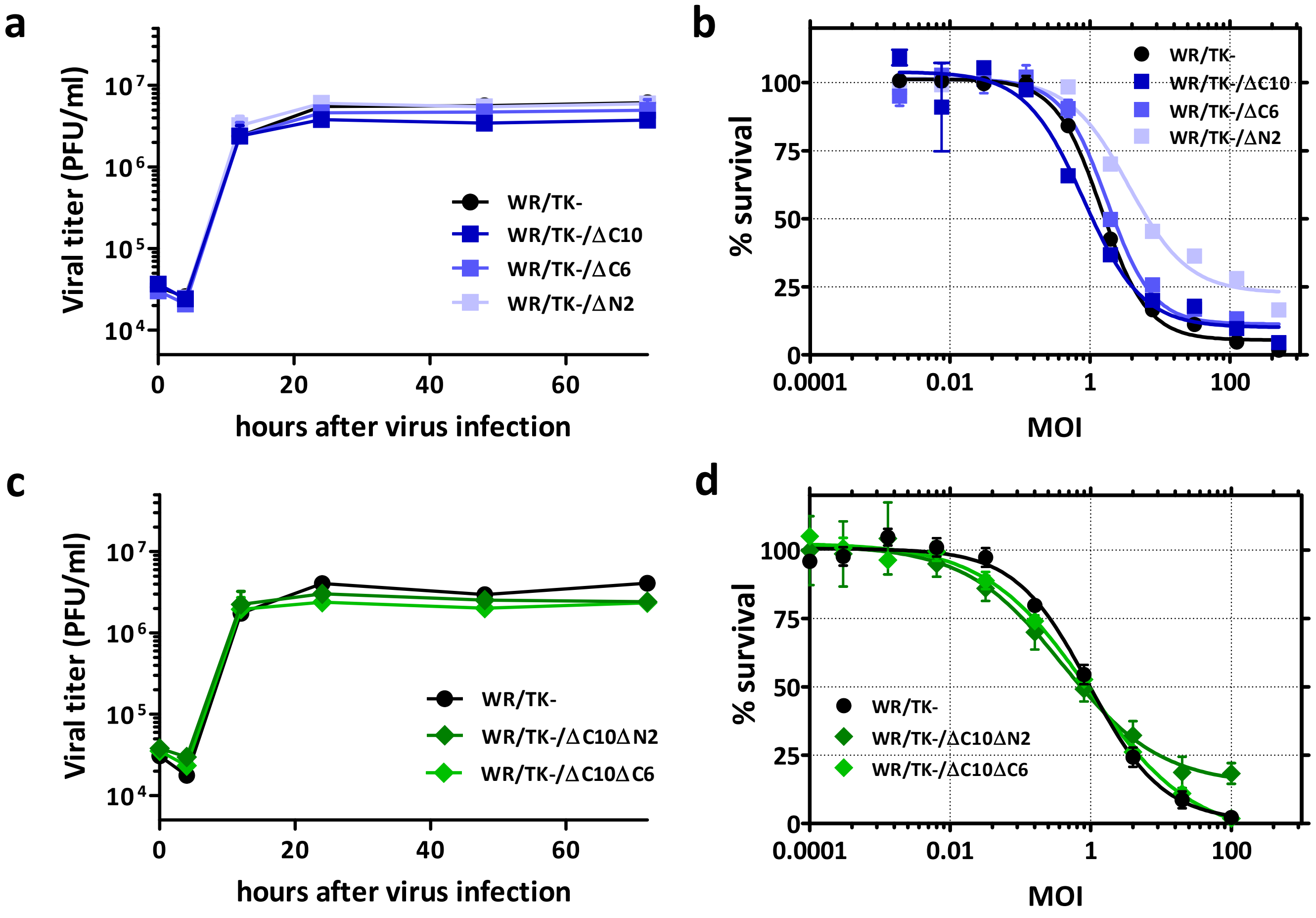

Supplementary Figure 1. Replication and cytotoxicity of various deletion mutant VACV. (a) Viral production of single-gene deleted viruses in HeLa cells. HeLa cells were infected with a multiplicity of infection (MOI) of 5 and, at indicated time points, samples were collected and viral titers were determined by plaque-assay. (b) Comparative cytotoxicity of single-gene deleted viruses in HeLa cells. HeLa cells were infected with indicated viruses at doses ranging from 200 to 0.001 PFU/cell. After 72 hours, % of cell death was evaluated. (c) Productive growth of double-gene deleted viruses in HeLa cells. HeLa cells were infected and treated as in (a) with indicated viruses. (d) Comparative cytotoxicity of double-gene deleted viruses in HeLa cells. HeLa cells were infected and treated as in (b) with indicated viruses.

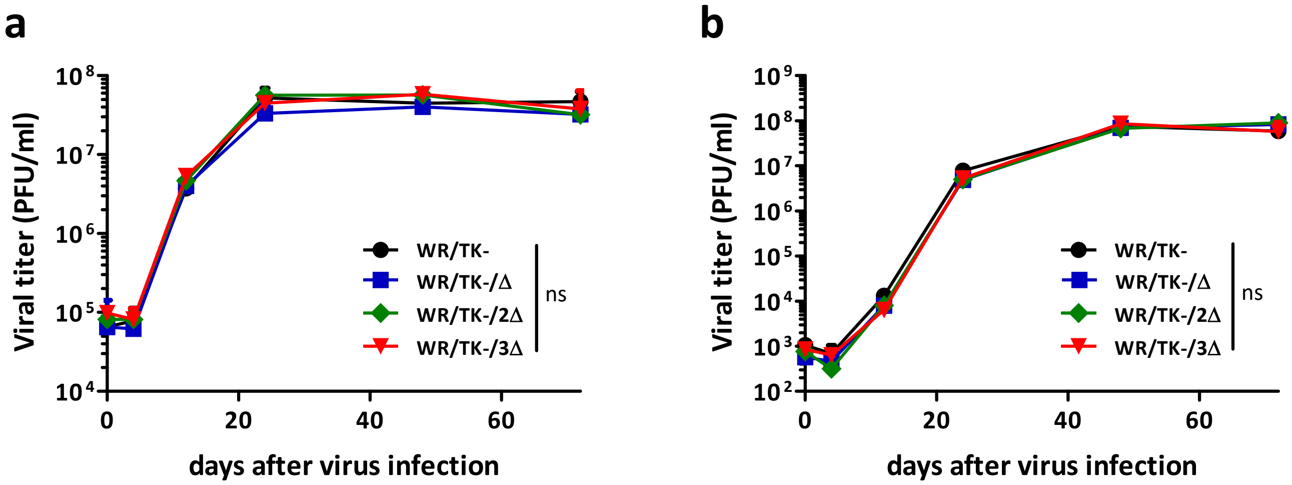

Supplementary Figure 2. Viral progeny production in A549 cells. A549 cells were infected with a multiplicity of infection (MOI) of 5 (a) or 0.05 (b) and, at indicated time points, samples were collected and viral titers were determined by plaque-assay. ns: not significant.

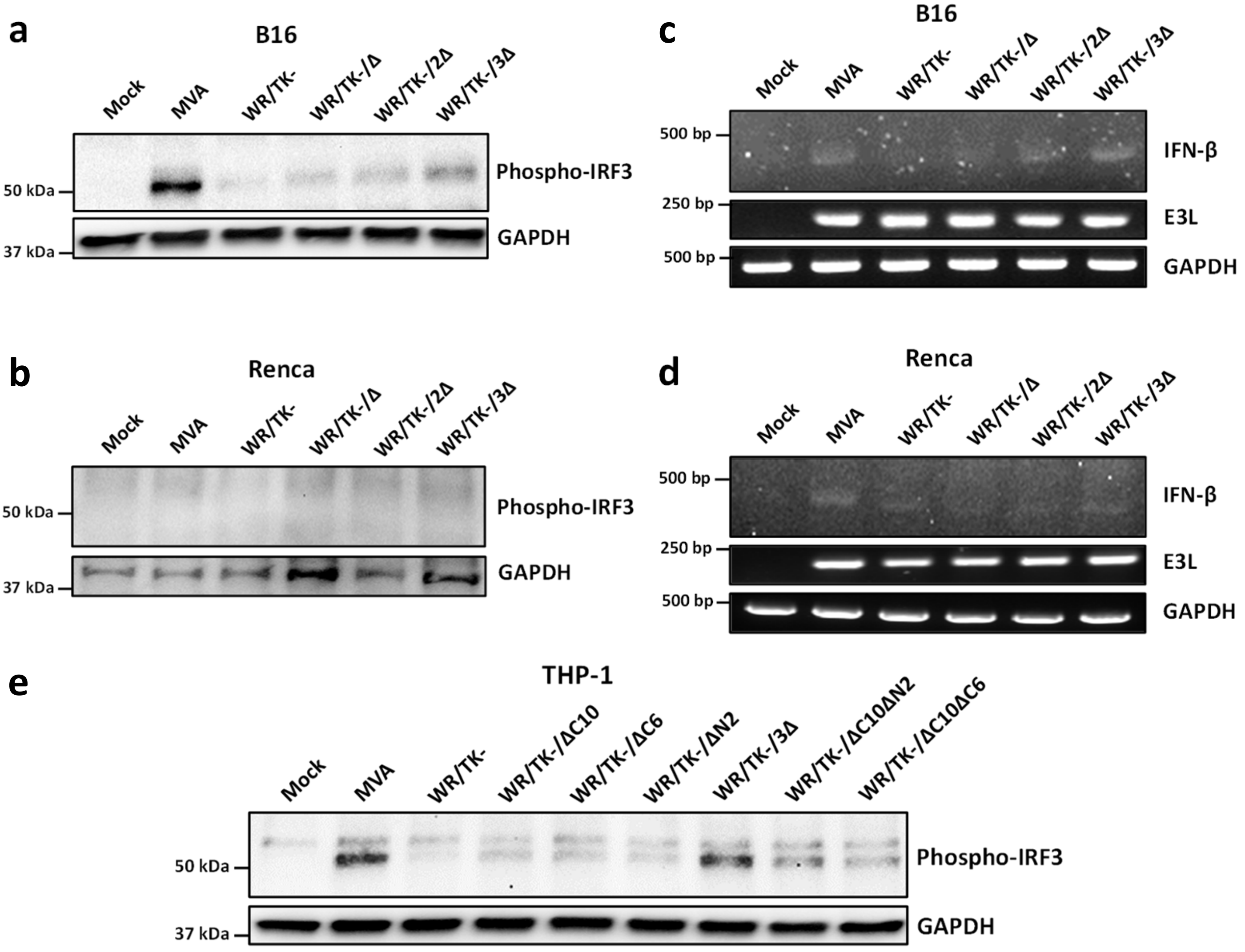

Supplementary Figure 3. Accumulation of genomic deletions is needed for activation of IRF3. (a-b) Phosphorylation of IRF3 after infection of mouse tumor cells. B16 (a) or Renca (b) cells were infected at a MOI of 10 and, 5 hours after infection, cells were lysed and Western Blot analysis was performed using a monoclonal antibody against phosphor-IRF3. MVA (Modified Vaccinia virus Ankara) served as a positive control and GAPDH-specific immunoblotting as a loading control. (c-d) Detection of IFN-β mRNA by RT-PCR after infection of mouse tumor cells. B16 (c) or Renca (d) cells were infected at a MOI of 5 and, 6 hours later, total RNA was obtained and the indicated mRNA of the indicated genes were amplified by RT-PCR. The VACV E3L mRNA was used as an infection control and GAPDH mRNA as a loading control. (e) Phosphorylation of IRF3 after infection with oncolytic viruses including all the possible combinations of target gene deletions. Human THP-1 cells were infected with indicated deletion mutant viruses at a MOI of 10 and, 5 hours after infection, cells were lysed and Western Blot analysis was performed using a monoclonal antibody against Phospho-IRF3. Vaccinia MVA strain was used as a positive control and GAPDH-blotting as a loading control.

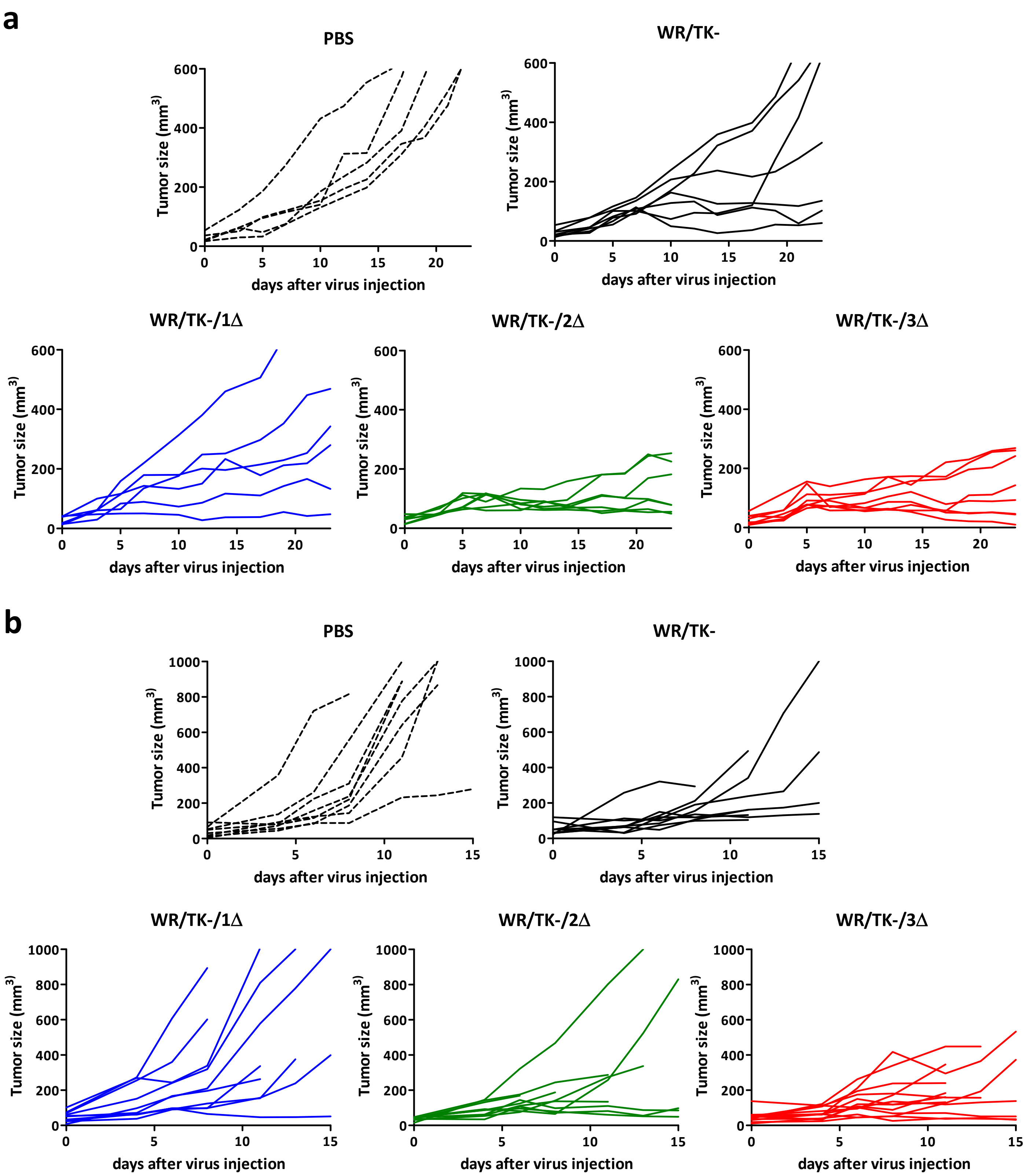

Supplementary Figure 4. Tumor growth curves of individual animals treated with candidate oncolytic VACV. Mean of treatments are shown in Figure 5. In short, mice with subcutaneous Renca (a) or B16 (b) tumors were treated by intratumoral injection with two doses of $1x10^7$ PFU of indicated viruses and tumor growth was monitored. Tumor growth of individual mice are depicted.
