# Supplementary material for: Activation of interferon regulatory factor 3 by replication-competent vaccinia viruses improves antitumor efficacy mediated by T cell responses
Source: Mol Ther Oncolytics. 2021 Jun 4;22:399–409. doi: 10.1016/j.omto.2021.06.001 (PMC8430050; doi:10.1016/j.omto.2021.06.001)
Supplement: Document S2. Article plus supplemental information [file mmc2.pdf]

Molecular Therapy
Oncolytics
Original Article

Activation of interferon regulatory factor 3 by replication-competent vaccinia viruses improves antitumor efficacy mediated by T cell responses

Stephanie Riederer,[1] Robert Fux,[1] Michael H. Lehmann,[1] Asisa Volz,[1,2] Gerd Sutter,[1,2] and Juan J. Rojas[1,3]

[1]Division of Virology, Department of Veterinary Sciences, LMU Munich, 80539 Munich, Germany; [2]German Center for Infection Research (DZIF), Partner Site Munich, 80539 Munich, Germany; [3]Department of Pathology and Experimental Therapies, IDIBELL, University of Barcelona, 08907 L'Hospitalet de Llobregat, Spain

Recently, oncolytic vaccinia viruses (VACVs) have shown their potential to provide for clinically effective cancer treatments. The reason for this clinical usefulness is not only the direct destruction of infected cancer cells but also activation of immune responses directed against tumor antigens. For eliciting a robust antitumor immunity, a dominant T helper 1 (Th1) cell differentiation of the response is preferred, and such polarization can be achieved by activating the Toll-like receptor 3 (TLR3)-interferon regulatory factor 3 (IRF3) signaling pathway. However, current VACVs used as oncolytic viruses to date still encode several immune evasion proteins involved in the inhibition of this signaling pathway. By inactivating genes of selected regulatory virus proteins, we aimed for a candidate virus with increased potency to activate cellular antitumor immunity but at the same time with a fully maintained replicative capacity in cancer cells. The removal of up to three key genes (*C10L*, *N2L*, and *C6L*) from VACV did not reduce the strength of viral replication, both *in vitro* and *in vivo*, but resulted in the rescue of IRF3 phosphorylation upon infection of cancer cells. In syngeneic mouse tumor models, this activation translated to enhanced cytotoxic T lymphocyte (CTL) responses directed against tumor-associated antigens and neo-epitopes and improved antitumor activity.

INTRODUCTION

During the last two decades, the understanding of the relationship between cancer and the immune system has considerably changed, and implemented the role of the immune system controlling tumorigenesis and tumor progression.[1,2] Cancer immunotherapies aim to mobilize the immune system to kill cancer cells and represent a revolution in cancer therapeutics: the field has seen outstanding progress in the last decade, showing unprecedented clinical responses. However, challenges arise for immunotherapies when treating a larger range of cancer types, mostly due to the complexity of the immune contexture and varying tumor immunogenicity.[3,4] Antibody-mediated blockade of immune checkpoints[5–7] and chimeric antigen receptor (CAR) T cells[8–10] have spearheaded this revolution, but a large number of novel modalities are being developed showing potential in preclinical and clinical trials. Further improvement of these novel immunotherapies is urgently needed to achieve clinical responses in currently resistant tumors.

Virotherapy uses genetically modified or naturally occurring viruses for the lysis of tumor cells. However, clinical data have demonstrated that these so-called oncolytic viruses primarily act as immunotherapies: viral replication serves as an extremely potent danger signal for the immune system within the tumor, allows overcoming tumor immunosuppression, and induces cytotoxic T lymphocyte (CTL) responses targeting a multitude of tumor antigens released by viral replication.[11,12] Among the various candidate viruses, vaccinia virus (VACV), the prototype human live virus vaccine used to eradicate smallpox,[13] displays several advantages as an oncolytic vector, including a fast and lytic replication cycle, an excellent human safety record, and high capacity for harboring transgenes.[14] Importantly, VACVs are highly immunogenic, and the targeted deletion of VACV genes can further improve this immunogenicity, transforming VACVs into useful tools for activating potent antitumor immune responses.[15]

Robust anti-tumor CTL responses have been shown to play a key role in the successful treatment of cancer.[16] In cancer vaccination, the use of the Toll-like receptor 3 (TLR3) agonist polyinosinic-polycytidylic acid (poly(I:C)) as an adjuvant has been shown to increase the number of CTLs targeting tumor antigens.[17] TLR3 activation leads to interferon (IFN) regulatory factor 3 (IRF3) phosphorylation and consequently to expression of type I IFNs, which correlates directly with increased levels of CTLs.[18–20] Interestingly, type I IFNs also play a crucial role in anti-VACV defense; that is, VACVs encode several proteins (including C10, A46, N2, or C6) that antagonize the TRL3-IRF3 signaling pathway at different levels.[21] As a result, phosphorylation of IRF3 is efficiently inhibited in VACV-infected cells, but deletions in some of these genes, such as C6 or N2, proved to improve CD8 T cell responses in vaccination strategies.[22,23] In addition, MVA (modified VACV Ankara), a highly attenuated strain of VACV with genomic mutations and deletions that inactivate many immunomodulatory

Received 17 December 2020; accepted 1 June 2021;
https://doi.org/10.1016/j.omto.2021.06.001.

Correspondence: Gerd Sutter, Division of Virology, Department of Veterinary Sciences, LMU Munich, Veterinärstrasse 13, 80539 Munich, Germany.
E-mail: gerd.sutter@lmu.de

This is an open access article under the CC BY license (http://creativecommons.org/licenses/by/4.0/).

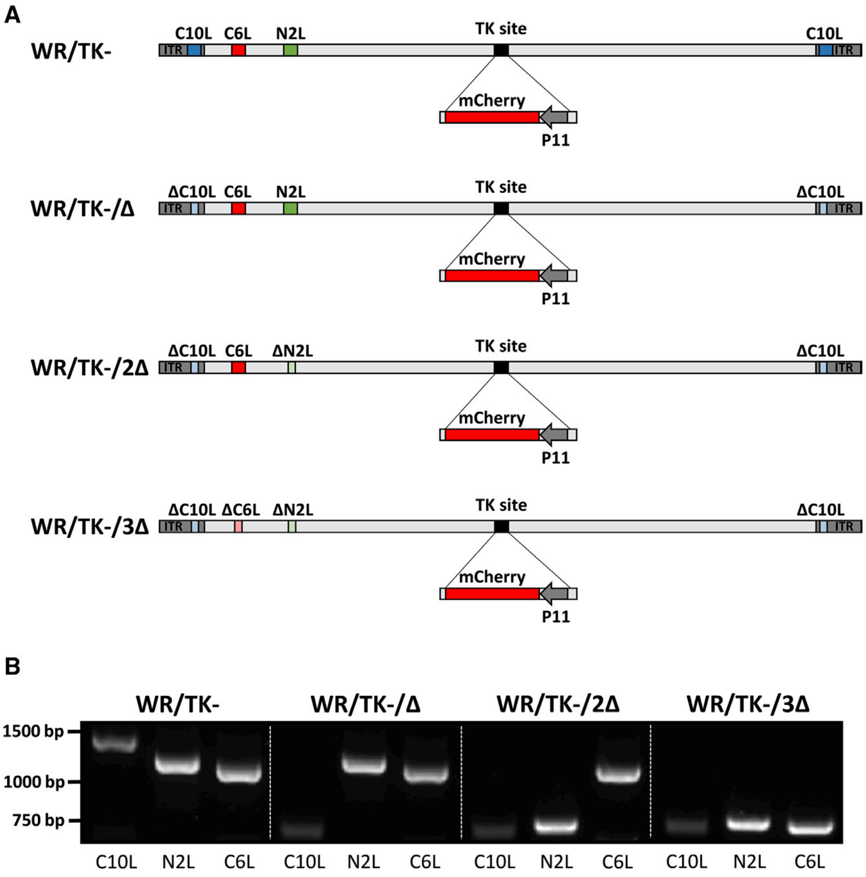

Figure 1. Generation of oncolytic VACV with deletions in key genes blocking activation of the IRF3 signaling pathway

(A) Schematic diagram of VACV genomes indicating the positions of the viral genes targeted by sequential deletion. For the prospect of monitoring viral replication, an expression cassette encoding the red fluorescent marker protein mCherry was inserted into the thymidine kinase (J2R) site of the virus genomes. (B) PCR analysis to confirm deletions in target genes. Expected size of the PCR products are as follows: *C10L* = 1,311 bp, Δ*C10L* = 692 bp; *N2L* = 1,126 bp, Δ*N2L* = 670 bp; *C6L* = 1,083 bp, Δ*C6L* = 682 bp.

genes, is able to robustly induce the secretion of type I IFN after infection.[24,25] However, the particular genetics of MVA are associated with a defective replication in mammalian cells, which greatly reduces its capacity for use as an oncolytic agent.[26–29] Thus, the generation of oncolytic VACV combining the capacity to activate the TLR3-IRF3 pathway with an efficient replication in cancer cells represents a major step toward an efficient VACV-based oncolytic therapy.

In this study, we constructed a battery of oncolytic VACVs by combining deletions in key VACV genes involved in the inhibition of IRF3 activation. We evaluated their replication competence in cancer cells as well as their ability to elicit T cell responses against tumor neo-antigens, demonstrating the feasibility to obtain replication-efficient VACVs with an increased capacity to activate the IRF3 pathway. Importantly, these modifications translated into improved treatments in mouse tumor models.

RESULTS

Generation of oncolytic VACV with deletions in key genes blocking activation of the IRF3 pathway

To improve cellular immune responses, we modified the candidate oncolytic VACV WR/TK−[30] (Western Reserve strain of VACV with a deleted thymidine kinase gene) by inactivating a set of viral genes involved in interfering with the IRF3 signaling pathway. Three genes were selected for deletion: *C6L*, *N2L*, and *C10L*. C6 interacts with the scaffold proteins NAP1, TANK, and SINTBAD;[31,32] N2 inhibits nuclear IRF3;[33] and C10 (named C16 in the WR strain) inhibits DNA-PK-mediated DNA sensing.[34,35] These genes were sequentially deleted, and Figure 1A schematically depicts deletions present in the genomes of the viruses tested in this study (WR/TK−/Δ, WR/TK−/2Δ, and WR/TK−/3Δ). Correct genetic modifications of the viral genomes were confirmed by PCR analysis with oligonucleotide primers flanking the deletion sites (Figure 1B) and by sequencing. In addition, the whole genome of WR/TK−/3Δ was sequenced, confirming that no further mutations could have affected the oncolytic capacity of the virus.

Deletion of genes blocking the IRF3 pathway do not interfere with oncolytic VACV *in vitro* features

Maintaining an efficient replication of the vector virus in cancer cells is important for achieving an effective oncolytic activity. Therefore, we evaluated whether the deletions or the combinations of deletions in the viral genomes have an influence on VACV replication in human cancer cells. For one-step growth or multiple-step growth analysis, we infected HeLa cells with candidate viruses at multiplicities of infection (MOIs) of 5 or 0.05 and, at indicated time points, cultures were harvested to determine viral titers by plaque assay. Both under one-step growth (Figure 2A; Figures S1A or S1C) or multiple-step growth conditions (Figure 2B), all candidate viruses replicated to titers similar to those obtained with the parental WR/TK− virus. In addition, replication was tested in A549 cells, a tumor cell line described to retain intact IFN pathways.[36,37] Again, the triple deletion mutant virus combining *C10L*, *N2L*, and *C6L* gene deletions efficiently grew in this cancer cell line, showing no difference in viral progeny production compared to the WR/TK− control virus (Figure S2).

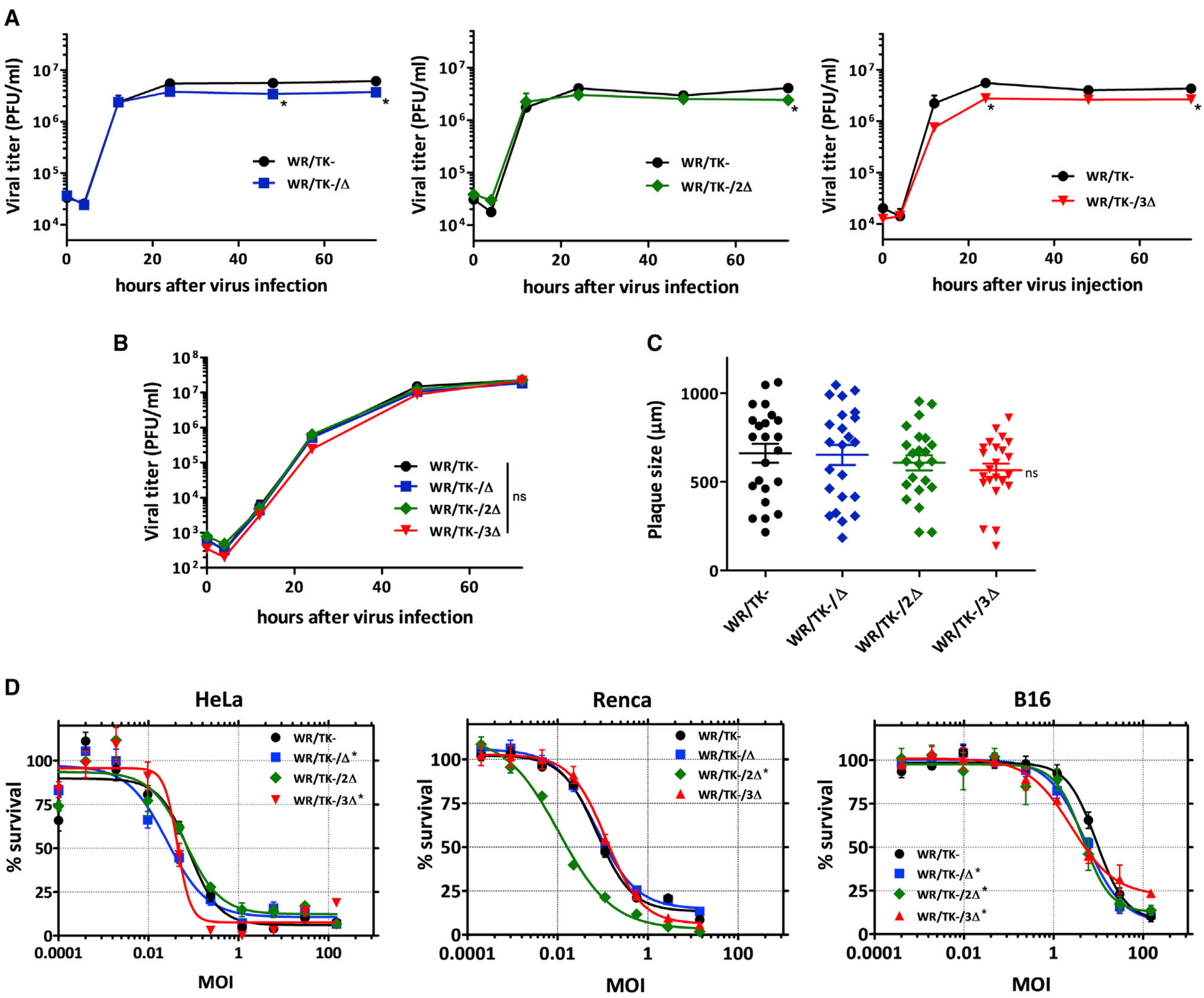

Figure 2. Unimpaired replication and capacity to kill cancer cells of mutant VACV accumulating deletions in genes inhibiting the IRF3 signaling pathway
(A and B) Productive multiplication of deletion mutant viruses. HeLa cells were infected with a multiplicity of infection (MOI) of 5 (A) or 0.05 (B) and, at indicated time points, samples were collected and viral titers were determined by a plaque assay. Virus yield was evaluated in quadruplicate. (C) Plaque size analysis in MA104 cells. MA104 cell monolayers were infected at an MOI of 0.05 and, at 72 h post-infection, stained with crystal violet solution before the diameter of plaques was measured. The diameter size (μm) of 25 representative plaques per virus and mean ± SD are depicted. (D) Comparative cytotoxicity in human and mouse tumor cell lines. Cells were infected with indicated viruses at doses ranging from 200 to 0.0001 PFU/cell. After 72 h, the percentage of killed cells was determined. $^{*}p < 0.05$ versus WR/TK−. ns, not significant.

The size of virus plaques formed in cell monolayers after infection can serve as an indicator of the viral capacity to destroy target cells upon propagation. In MA104 cells, a cell line supporting the formation of distinct plaque lesions upon VACV infection, the plaques obtained after infection with the candidate viruses were not significantly different in size compared to those formed after infection with WR/TK−, although we observed a tendency for plaque size reduction with the accumulation of genomic deletions (Figure 2C).

To confirm the unimpaired capacity of candidate oncolytic viruses to kill cancer cells, we assessed their efficacy in destroying tumor cells by a metabolic assay. We infected both human (HeLa) and mouse cancer cell lines (Renca and B16) with different MOIs (ranging from 0.0001 to 200) and, 72 h after infection, the remaining metabolic activity of cells was determined (Figure 2D; Figures S1B and S1D). The capacity to kill cancer cells was not affected by the accumulation of gene deletions and resulted in very similar patterns of cell death for infections with WR/TK−/Δ, WR/TK−/2Δ, and WR/TK−/3Δ compared to the parental virus WR/TK−.

Deletion of viral genes interfering with the IRF3 signaling pathway leads to phosphorylation of IRF3 and expression of IFN-β

To evaluate whether infection with candidate oncolytic VACV (WR/TK−/Δ, WR/TK−/2Δ, and WR/TK−/3Δ) leads to activation of the

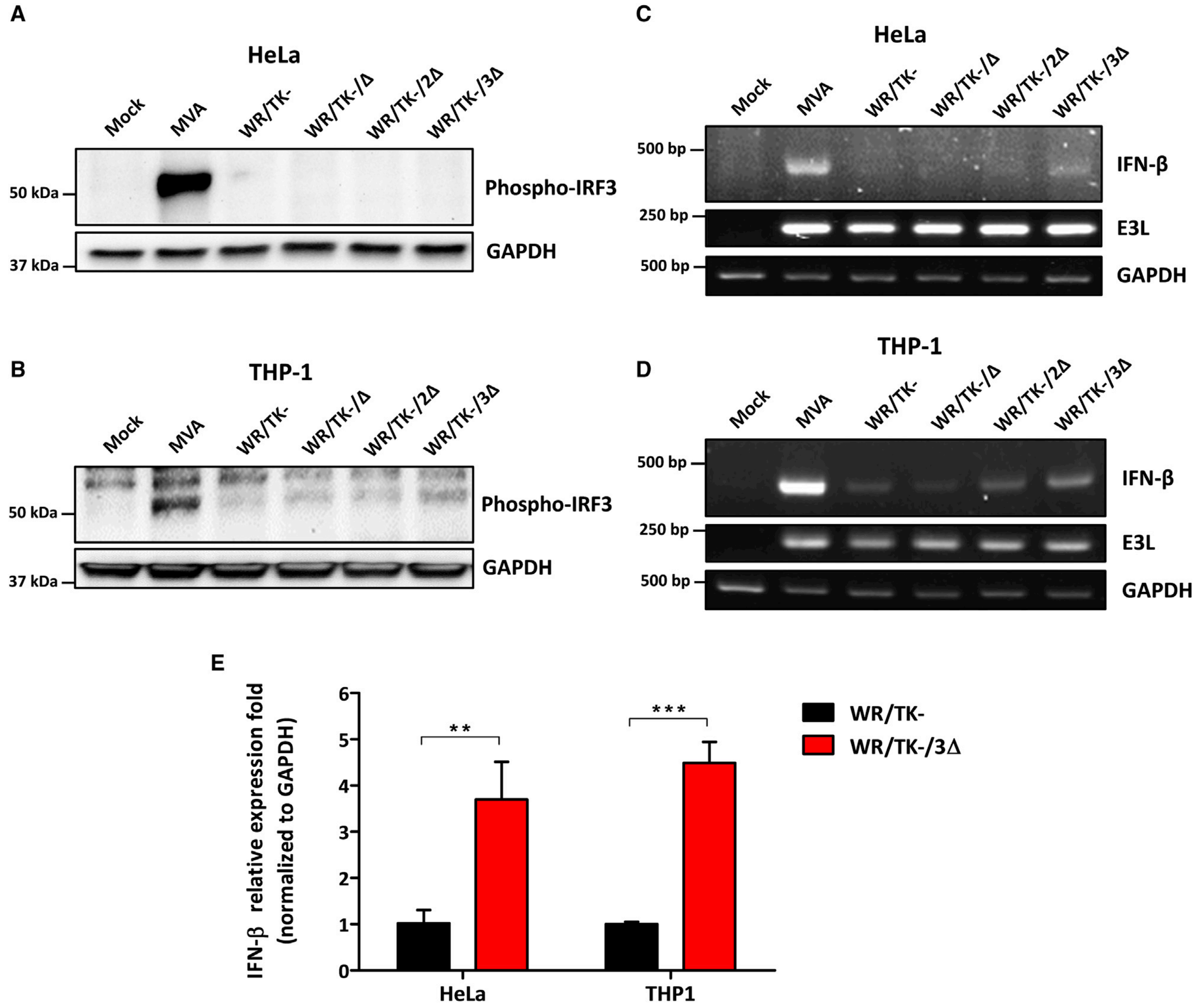

Figure 3. Activation of the IRF3 pathway by candidate oncolytic VACV

(A and B) Deletion of viral genes interfering in the IRF3 pathway leads to IRF3 phosphorylation in human cells. HeLa (A) and THP-1 (B) cells were infected at an MOI of 10 and, 5 h after infection, cells were lysed and western blot analysis was performed using a monoclonal antibody against p-IRF3. The non-replicating VACV MVA (modified vaccinia virus Ankara) served as a positive control and GAPDH-specific immunoblotting served as a loading control. (C and D) Detection of IFN-β mRNA by RT-PCR in human cells. HeLa (C) and THP-1 (D) cells were infected at an MOI of 5. At 6 h after infection, total RNA was obtained and mRNAs of indicated genes were amplified by RT-PCR. The VACV E3L mRNA was used as an infection control, and GAPDH mRNA was used as a loading control. (E) Relative expression of IFN-β measured by qRT-PCR. Indicated cells were infected with WR/TK− or WR/TK−/3Δ, and total RNA was obtained as indicated in (C) and (D). mRNA expression levels of IFN-β were determined via qRT-PCR and normalized to that of the *GAPDH* gene by the $2^{-\Delta\Delta Ct}$. $^{**}p < 0.01$, $^{***}p < 0.001$.

IRF3 pathway, we determined the amounts of phosphorylated IRF3 (p-IRF3) by western blot after infection of human cancer cells. As a positive control for the activation of the IRF3 pathway, we used infections with the replication-deficient MVA, which is a natural VACV mutant with many inactivated viral genes and is known to efficiently activate IRF3.[38] In HeLa cells, levels of p-IRF3 were not increased by the presence of deletions compared to the parental virus WR/TK− (Figure 3A). However, in THP-1 cells (a human monocyte cell line broadly used for immune assay experiments, Figure 3B) we detected increasing amounts of p-IRF3 upon infection with viruses harboring accumulating inactivation in genes interfering with the IRF3 pathway. In addition, phosphorylation of IRF3 after infection of mouse cancer cell lines was also evaluated. In B16 cells (Figure S3A), a clear tendency to detect increasing levels of p-IRF3 was observed with accumulation of deletions, similar to THP-1 cells. In Renca cells, no phosphorylated protein was detected even for the MVA-positive control (Figure S3B). Effects of single gene deletions were discarded by western blot analysis of extracts from THP1 cells infected with

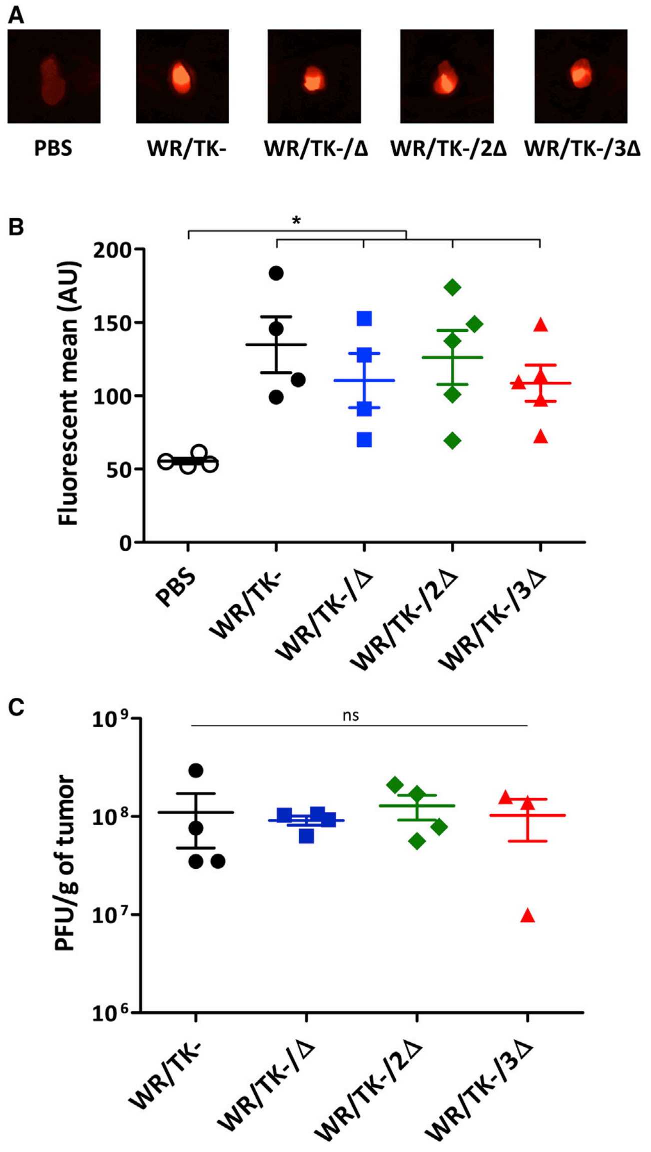

Figure 4. Replication of deletion mutant VACV in tumor models *in vivo*

5×10^5 Renca cells were subcutaneously implanted on the flank of 6- to 8-week-old BALB/c mice (n = 4–5). At day 0, a dose of 1×10^7 PFU was intratumorally injected and, 4 days later, mice were sacrificed and tumors were harvested. (A) Images of representative tumors showing mCherry-specific fluorescence. (B) Tumor fluorescence quantified using a MacroImaging system. Fluorescence of individual tumors and group means ± SD are shown. (C) Viral titers determined by plaque assay after tumor homogenization. Titers obtained from each independent tumor and means ± SD are depicted. *$p < 0.05$. ns, not significant.

mutant viruses, including all possible combinations of *C10L*, *N2L*, and *C6L* gene deletions (Figure S3E).

IRF3 activation was confirmed by RT-PCR. We detected increased levels of IFN-β mRNA upon infection with the WR/TK−/3Δ virus (Figures 3C and 3D; Figures S3C and S3D). Of note, this finding includes HeLa and Renca cells (Figure 3D; Figure S3D), where increased levels of p-IRF3 protein could not be detected by immunoblot analysis. Quantification of IFN-β mRNA by qRT-PCR demonstrated a significant increase mediated by the WR/TK−/3Δ virus compared to the levels activated by the WR/TK− control virus (Figure 3G).

Replication of deletion mutant viruses is not impaired in mouse tumor models

To ensure that virus replication remains unimpaired *in vivo*, we injected mice bearing Renca tumors (mouse renal adenocarcinoma) intratumorally with the candidate deletion mutant viruses and, 4 days after virus injection, viral growth was evaluated. Taking advantage of mCherry co-expression (Figure 1A), fluorescence emitting from tumor tissues was quantified (Figures 4A and 4B). In addition, we titrated the virus loads within tumors (Figure 4C). Both methodological approaches showed that deletion mutant viruses and the parental virus WR/TK− replicated to very similar levels in tumor tissues. This indicates that deletion of up to three genes interfering with the IRF3 pathway does not hinder effective VACV replication, both *in vitro* and *in vivo*.

Improved antitumor activity of oncolytic candidate VACV

As a next step, we evaluated the antitumor efficacy of the deletion mutant VACV *in vivo* using intratumoral virus delivery in two syngeneic mouse tumor models: BALB/c mice bearing Renca tumors and C57BL/6 mice bearing B16 tumors. In the Renca model, the injection of WR/TK−/2Δ or WR/TK−/3Δ viruses resulted in a strong significant reduction of tumor growth in comparison to the therapeutic effect observed with the parental WR/TK− (Figure 5A; Figure S4A). Additionally, we also observed an increased survival time of mice injected with double and triple deletion mutant VACV (Figure 5B). When tested in the mouse melanoma tumor model B16, and although some mice needed to be sacrificed early in the experiment due to tumor ulceration (Figure S4B), the WR/TK−/3Δ virus was able to induce a significant reduction in tumor growth (Figure 5C), but this was not the case with the WR/TK−/2Δ virus. The survival time of this model is shown in Figure 5D. MVA was not included as a control in these experiments, as the antitumor activity of this virus is limited by its incapacity to productively replicate in tumor cells when compared to WR/TK−.[26]

Induction of tumor-specific cellular immune response by deleted VACVs

Our hypothesis was that increased antitumor activity of deleted VACV is mediated by increased type I IFN levels within tumors, which leads to a more robust cellular antitumor immunity. Thus, we tested the IFN-β concentration within tumors by ELISA 4 days after virus administration. We detected a 2-fold increase in IFN-β levels in tumors injected with WR/TK−/3Δ compared to those injected with WR/TK−, although the difference was not significant (Figure 6A). Finally, we evaluated the tumor epitope-specific T cell responses established following virus administration in the B16 tumor model. Enzyme-linked immunospot (ELISPOT) assays were performed to determine the T cell response directed against the virus

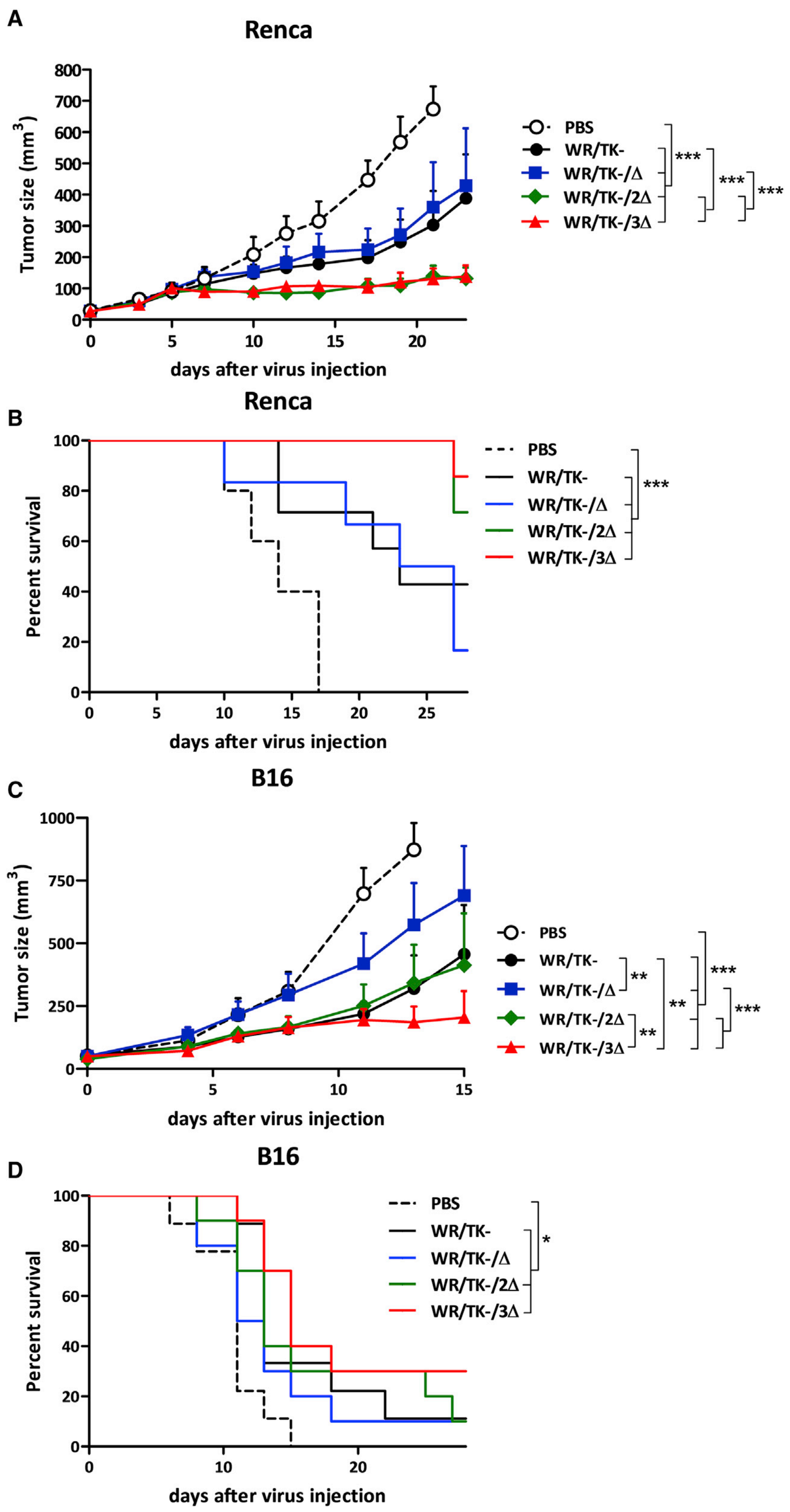

Figure 5. Increased *in vivo* antitumor activity of candidate oncolytic VACV with combination of gene deletions rescuing IRF3 activation

(A–D) 5×10^5 tumor cells were subcutaneously implanted at day −9 on the flank of 6- to 8-week-old BALB/c mice (Renca tumors, A and B) or C57BL/6 mice (B16 tumors, C and D), and viruses were intratumorally administered at days 0 and 4 at a dose of 1×10^7 PFU/injection. PBS-injected mice served as controls. For monitoring tumor growth, the tumors were measured two to three times per week until termination criteria were reached. Tumor volume (A and C) and overall survival (B and D) are plotted for 7–9 mice/group ± SEM. *$p < 0.05$, **$p < 0.01$, ***$p < 0.001$.

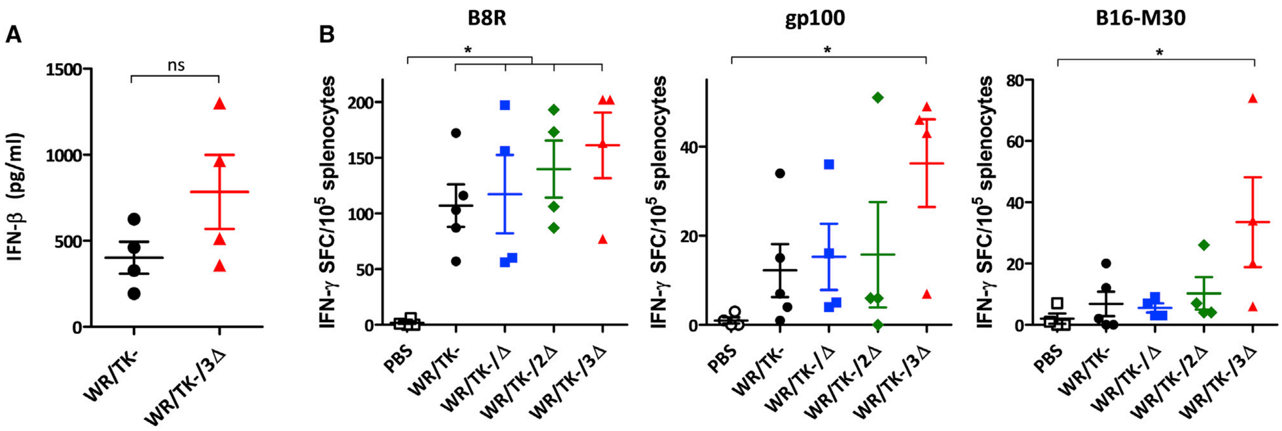

Figure 6. Intratumoral administration of deletion mutant VACV induces antitumor T cell responses directed against tumor neo-antigens
(A) Intratumoral IFN-β concentrations after mutant VACV injection. BALB/c mice harboring Renca tumors were treated with a single intratumoral dose of 1×10^7 PFU and, 4 days later, tumors were harvested and homogenized. IFN-β concentrations were determined by an ELISA. (B) Cellular immune response evaluated by an IFN-γ ELISPOT assay. C57BL/6 mice harboring B16 tumors were treated as indicated in Figure 5, and, 8 days after virus administration, splenocytes were prepared, *in vitro* stimulated with indicated peptides, and analyzed for IFN-γ-producing cells by ELISPOT. Individual values of IFN-γ spot forming cells (SPC)/10^5 splenocytes in 4–5 mice/group and mean ± SD are plotted. *$p < 0.05$. ns, not significant.

(immunodominant VACV-specific B8R peptide epitope),[39] a non-mutated gp100 tumor-associated antigen epitope,[40] and the tumor neo-epitope B16-M30.[41] The injection of WR/TK−/3Δ increased T cell reactivity to all the three epitopes (Figure 6B); however, of note, we found clearly increased levels of epitope-specific IFN-γ-producing T cells directed against the tumor antigens (gp100 and B16-M30) compared to treatments with the parental virus WR/TK−.

DISCUSSION

Our goal in this work was to obtain a fully replication-competent oncolytic VACV with improved capacity to activate antitumor T cell responses. The replication of oncolytic VACV in cancer cells leads to the release of danger-associated molecular patterns (DAMPs) together with a multitude of tumor-specific antigens, turning "cold" tumors into "hot" tumors for more efficacious immunotherapy.[42] We based our strategy for improving antitumor T cell responses on the observation that poly(I:C), used as an adjuvant in cancer vaccination, leads to T helper 1 (Th1) polarization,[43] which directly correlates with robust antitumor immunity in the clinic.[44] As poly(I:C) selectively activates TLR3, we attempted to construct an oncolytic vector virus with the capacity to activate the TLR3-IRF3 pathway after infection.

In line with its outstanding capacity to evade antiviral innate immunity, VACV encodes for several immunomodulatory proteins directly interfering with the host TLR3-IRF3 innate response pathway. In order to promote the activation of this pathway, we constructed a series of oncolytic VACVs combining the deletion of the thymidine kinase gene (to achieve selective replication in cancer cells) with targeted inactivation of selected genes interfering with IRF3 pathway activation. We chose three target proteins due to their important inhibitory mechanisms at different levels in the pathway: C10 (also known as C16 due to its nomenclature in the Western Reserve strain) prevents double-stranded DNA (dsDNA) recognition by DNA-PK;[34,35] N2 interferes by yet unknown mechanisms downstream of p-IRF3 and its nuclear translocation;[33] and C6 interacts with NAP1, TANK, and SINTBAD, the scaffold adaptor proteins for the kinases TBK1 and IKKε, which lead to IRF3 activation.[31,32] We constructed all of the possible mutant VACVs combining deletions in these three genes, but one single-, one double-, and the triple-deletion mutant viruses were selected for complete testing; the selection was performed based on the lack of loss in cytotoxicity and the replicative capacity *in vitro* (Figure S1). Deletions included in the final candidate oncolytic VACV are depicted in Figure 1. As shown in Figure 2 and Figure S2, inclusion of up to three of these mutations does not impair the capacity of VACV to replicate in cancer cells or their cancer cell-killing efficacy *in vitro*.

Combination of deletions in *C10L*, *N2L*, and *C6L* genes results in activation of the TLR3-IRF3 pathway as demonstrated by detection of p-IRF3 and IFN-β mRNA (Figure 3; Figure S3). Increasing levels were detected by the introduction of deletions to the genome of the control virus WR/TK−, and Figure S3E shows that this activation is mediated by this accumulation of deletions rather than by any of the single deletions. In mouse models, this TLR3-IRF3 pathway activation translates into increased intratumoral IFN levels (Figure 6A) and improved T cell responses, both directed against the virus and the tumor (Figure 6B). Importantly, anti-tumor T cells are directed against tumor-associated antigens (gp100) but also against tumor neo-epitopes (B16-M30), and T cell activities elicited by WR/TK−/3Δ are significantly higher as compared with responses obtained with the non-treated group. Finally, these enhanced tumor-directed immune responses are associated with an improved antitumor activity in two syngeneic mouse tumor models (Figure 5), strongly suggesting the feasibility and efficacy of the proposed strategy.

Previously, an oncolytic VACV expressing TRIF (the main adaptor in the TLR3-IRF3 signaling pathway) also explored the strategy of activating the TLR3-IRF3 pathway after infection of tumor cells.[30] This virus demonstrated a switch from a Th2- to a Th1-skewed response and displayed enhanced therapeutic activity in mouse models. However, replication of the virus was strongly hindered within tumors (using the Renca model) due to massive pathway activation; conversely, our novel strategy of accumulating up to three deletions in VACV genes interfering with the TLR3-IRF3 pathway fully conserved the replication capacity in Renca tumors (Figure 4). We have previously demonstrated the importance of VACV replication for activating an antitumor immune response,[26] which is discrepant to some previous reports.[45] Yet, virus replication leads to tumor cell lysis and release of tumor antigens, in addition to amplifying the initial dose administered and multiplying danger signals. Thus, maintaining an efficient replication in tumor cells is a key factor for the outcome of oncolytic therapies and should be an important feature when developing a candidate for clinical evaluation.

Although able to improve antitumor immune responses, levels of p-IRF3 and IFN-β mRNA detected after infection with WR/TK−/3Δ do not reach the levels observed after infection with MVA (Figure 3; Figure S3). Previously, VACV incorporating single deletions in the *C6L*, *N2L*, or *C10L* gene demonstrated enhanced immunogenicity and highly reduced virulence in mice.[22,33,46] However, we were unable to detect TLR3-IRF3 pathway activation *in vitro* after infection with any of these three single deleted VACVs (Figure S3E). Thus, activities detected in *in vitro* assays may not properly reflect levels of activation *in vivo* in animal models. Currently, we are working on incorporating further deletions into our WR/TK−/3Δ candidate virus to test the possibility to further improve this activation. Additional VACV regulatory proteins with inhibitory functions in activation of the TLR3-IRF3 signaling pathway include A46, which interacts with TRIF,[47] K7, which binds the DEAD-box RNA helicase 3 (DDX3),[48] and B19 (also known as B18 due to its nomenclature in the Western Reserve strain), which is a soluble type I IFN receptor.[49] Recently, the VACV *B2R* gene has been reported to encode a viral nuclease with cyclic guanosine monophosphate-AMP (cGAMP)-specific activity and an important role in the inhibition of the pathway.[50] However, further deletions incorporated to the WR/TK−/3Δ virus may compromise the ability of these deletion mutant viruses to efficiently replicate in cancer cells, as demonstrated by the growth deficiency of the natural deletion mutant virus MVA. An appropriate balance between the activation of danger signaling pathways and virus replication must be found for optimizing oncolytic VACV immunotherapies.

Taken together, the data presented herein demonstrate that it is possible to generate an oncolytic VACV with the ability to activate the TLR3-IRF3 pathway while maintaining full capacity to productively replicate in cancer cells. Importantly, the combination of these features translates into improved antitumor immunity and antitumor efficacy of the oncolytic vector virus. This strategy can be combined with other genetic modifications or immunotherapies to produce robust responses in patients suffering from a variety of solid tumors.

MATERIALS AND METHODS

Cell lines and viruses

All cell lines used in this research (MA104, HeLa, Renca, B16, and THP-1 cells) were obtained from the American Type Culture Collection (ATCC). Primary chicken embryo fibroblasts (CEFs) were prepared from 10-day-old chicken embryos (specific pathogen-free [SPF] eggs, VALO BioMedia, Cuxhaven, Germany). All cell lines were maintained in recommended culture media containing 5%–10% fetal bovine serum and antibiotics at 37°C, 5% CO_2.

All recombinant viruses used or constructed in this work, except for the MVA control virus, are based on the VACV strain Western Reserve. To enhance selective replication in cancer cells, VACV WR/TK− was constructed by inactivation of the viral thymidine kinase gene through insertion of an expression cassette for the *mCherry* reporter gene under transcriptional control of the VACV late promoter P11. VACV WR/TK− served as the backbone for deleting the target VACV genes blocking the IRF3 pathway activation. The *C6L*, *C10L*, and *N2L* genes were inactivated by homologous recombination replacing the original gene sequence with a synthetic construct containing two 350-bp DNA sequences upstream and downstream of the genomic site targeted for deletion. In addition, the start codon in the synthetic target gene sequence was mutated. For homologous recombination and generation of the VACV deletion mutants, a shuttle plasmid DNA containing the synthetic gene sequence (Δ*C6L*, Δ*C10L*, or Δ*N2L*) was transfected into MA104 cells that were infected with VACV WR/TK− 90 min prior to plasmid transfection. The deletion mutants were clonally isolated by a positive-negative selection system based on GFP as a reporter, and all genetic modifications were confirmed by PCR and sequencing. For the construction of mutant viruses with accumulating gene deletions, the homologous recombination process was repeated with a different shuttle plasmid once the first deletion was confirmed. Viruses were purified as previously described[26] and titrated by a plaque assay in MA104 cells (for replication-competent VACV) or in CEF cells (for the MVA strain). The whole-genome sequences of the WR/TK− and WR/TK−/3Δ viruses were determined and analyzed using MinION technology (Laboratory for Functional Genome Analysis, LMU, Germany).

Virus growth assay and plaque size

2×10^5 cells were seeded in 24-well plates and infected at an MOI (plaque-forming units [PFU]/cell) of 5 or 0.05. One hour after infection, cells were washed with PBS and new pre-warmed medium was added. At different time points (0, 4, 12, 24, 48, and 72 h after infection), samples were harvested and frozen at −80°C. Viral titer was determined by a plaque assay after three freeze-thaw-cycles.

To assess the size of the plaques formed by the different viruses, MA104 cells were infected at an MOI of 0.05 and, at 72 h post-infection, the diameter of plaques was measured after dying with crystal violet.

In vitro cytotoxicity assay

Cytotoxicity assays were performed by seeding 5×10^4 cells in 96-well plates. Cells were infected with 1:5 serial dilutions starting at an MOI

of 150 (ranging from 150 to 0.0001) and incubated at 37°C for 72 h. After 3 days, cells were checked for remaining metabolic activity using a non-radioactive cell proliferation assay (CellTiter 96 AQueous non-radioactive cell proliferation assay, Promega, Fitchburg, WI, USA) following the manufacturer's instructions.

Protein analysis

Indicated cells were seeded in 24-well plates and infected at an MOI of 10. 5 h after infection, cells were harvested and lysed using radioimmunoprecipitation (RIPA) assay buffer supplemented with 1% protease/phosphatase inhibitor cocktail (Thermo Scientific, Waltham, MA, USA). Protein extracts were quantified by a bicinchoninic acid (BCA) assay kit, and equal amounts of protein were separated by 10% SDS gel and transferred to a nitrocellulose blotting membrane. After blocking the membrane with Tris-buffered saline (TBS)/Tween 20 with 5% BSA, a monoclonal anti-p-IRF3 primary antibody (S396 rabbit monoclonal antibody [mAb], Cell Signaling Technology, Danvers, MA, USA) diluted 1:1,000 in TBS/Tween 20 with 1% BSA and a polyclonal anti-rabbit conjugated with horseradish peroxidase (HRP) (Cell Signaling Technology, Danvers, MA, USA) diluted 1:5,000 in TBS/Tween 20 with 1% BSA were used for detection. For the loading control, a rabbit anti-GAPDH antibody (Cell Signaling Technology, Danvers, MA, USA) diluted 1:1,000 in TBS/Tween 20 with 1% BSA was used.

mRNA expression analysis

1×10^6 cells/well were seeded in 24-well plates and infected at an MOI of 5. At 6 h post-infection, cells were harvested and total RNA was purified using a RNeasy Plus mini kit (QIAGEN, Hilden, Germany). To eliminate remaining genomic DNA, samples were digested with RQ1 RNase-free DNase (Promega, Madison, WI, USA). cDNA was synthesized using an Omniscript RT kit (QIAGEN, Hilden, Germany), and the PCR was performed on 1.5 μg of cDNA using specific primers for the mRNA of interest and run in an agarose gel.

For qRT-PCR analysis, cDNA samples were obtained as described above, and qRT-PCR was performed using the Luna universal qPCR master mix (New England Biolabs, Ipswich, MA, USA) under the following conditions: initial denaturation at 95°C for 1 min, followed by 42 cycles of 95°C for 15 s, 60°C for 30 s, followed by melting curve at 65°C–95°C, 0.5°C/cycle. The AriaMx real-time PCR system (Agilent Technologies, Santa Clara, CA, USA) and the corresponding Aria 1.7 software were used to perform and analyze the qPCRs. Relative expressions of IFN-β were normalized to those of GAPDH, using the $2^{-\Delta\Delta Ct}$ method.[51] The following primers, which were previously described,[52,53] were used: human (h)IFN-β forward, 5′-GCTTGGATTCCTACAAAGAAGCA-3′, reverse, 5′-ATAGATGGTCAATGCGGCGTC-3′; hGAPDH forward, 5′-ATTTGGCTACAGCAACAGG-3′, reverse, 5′-TTGAGCACAGGGTACTTTATT-3′.

Mouse models

All animal experiments were handled in compliance with the German regulations for animal experimentation (Animal Welfare Act, approved by the Government of Upper Bavaria, Munich, Germany). 6- to 8-week-old female BALB/c (Renca tumor model) or C57BL/6 (B16 tumor model) mice were purchased from Charles River Laboratories and housed in an isolated (ISO) cage unit with free access to food and water. Tumor cells for implantation were maintained *in vitro* at standard conditions. At the day of implantation, cells were trypsinized and 5×10^5 cells were implanted in the flank of the mice. When tumors reached a volume of 50–100 mm^3, mice were randomized and viruses were administrated intratumorally.

Study of viral replication and IFN-β quantification *in vivo*

Tumors were established as described above. After randomization of mice (n = 4–6), they received at day 0 a single intratumoral dose of 1×10^7 PFU. Mice were sacrificed at day 4 and tumors were harvested, washed with PBS, and the fluorescence signal from tumors was acquired using a GelDoc imaging system (Bio-Rad, Hercules, CA, USA) and quantified using ImageJ.

For determining viral titer and IFN-β concentrations within tumors, mice were treated as described above and sacrificed at day 4 after viral administration. Tumors were harvested, weighed, and homogenized using metal beads and a tissue homogenizer (QIAGEN, Hilden, Germany). Virus titers were determined by plaque assay on MA104 cells, and IFN-β was quantified using a mouse IFN-β ELISA kit (R&D Systems, Minneapolis, MN, USA).

In vivo antitumor activity

Tumors were established as described above. Mice were treated twice (days 0 and 4) with an intratumoral dose of 1×10^7 PFU of indicated viruses. Mice were monitored daily, tumors were measured 3 times per week using a caliper, and tumor volume was calculated as the length × width × height in mm^3. Mice were euthanized when tumors reached termination criteria.

IFN-γ ELISPOT

Tumors were established as described above, and mice were treated twice with an intratumoral dose of indicated viruses. 5 days after the second virus injection, mice were sacrificed and spleens harvested. 2×10^5 cells were cultured for 48 h in anti-IFN-γ (Mabtech, Stockholm, Sweden) pre-coated 96-well plates together with 2 μg/mL peptides. The synthetic peptides used for restimulation were B8R (TSYKFESV), gp100 (EGSRNQDWL), and B16-M30mut (PSKPSFQEFVDWENVSPELNSTD). An automated ELISPOT reader (A.EL.VIS Eli.Scan, Hannover, Germany) was used for counting and analyzing.

Statistical analysis

A standard Student's t test (two-tailed) was used for analyzing results in Figures 2 and 3G and Figure S1. A one-way ANOVA and Tukey's multiple comparison test was used for analyzing Figures 4 and 6. In Figure 5, a two-way ANOVA and Bonferroni posttest were chosen for analyzing tumor growth curves, and a log-rank test was used for survival curves. In all cases, significance was achieved when $p < 0.05$.

SUPPLEMENTAL INFORMATION

Supplemental information can be found online at https://doi.org/10.1016/j.omto.2021.06.001.

ACKNOWLEDGMENTS

We thank Patrizia Bonert, Ursula Klostermeier, Johannes Döring, and Axel Groβ for expert help in animal studies. We thank Marlowe Peters and Sylvia Jany for assistance and support with RT-PCR and ELISPOT assays. This work was supported by Deutsche Krebshilfe project no. 70112543 (to J.J.R. and G.S.), and by Spanish Ministry of Science I+D+i project no. PID2019-109102RA-I00 (to J.J.R.). J.J.R. is funded by the Spanish Ministry of Science through a Ramon y Cajal program (RYC2018-025425-I). The graphical abstract was created with BioRender.com.

AUTHOR CONTRIBUTIONS

Conceptualization, G.S. and J.J.R.; methodology, G.S., R.F., M.H.L., A.V., and J.J.R.; investigation, S.R., R.F., M.H.L., A.V., and J.J.R.; writing – original draft, S.R.; writing – review & editing, G.S. and J.J.R.; supervision, G.S. and J.J.R.; funding acquisition, G.S. and J.J.R.

DECLARATION OF INTERESTS

The authors declare no competing interests.

REFERENCES

1. Chen, D.S., and Mellman, I. (2013). Oncology meets immunology: The cancer-immunity cycle. Immunity *39*, 1–10.
2. Schreiber, R.D., Old, L.J., and Smyth, M.J. (2011). Cancer immunoediting: Integrating immunity's roles in cancer suppression and promotion. Science *331*, 1565–1570.
3. Yang, Y. (2015). Cancer immunotherapy: Harnessing the immune system to battle cancer. J. Clin. Invest. *125*, 3335–3337.
4. Nakamura, K., and Smyth, M.J. (2017). Targeting cancer-related inflammation in the era of immunotherapy. Immunol. Cell Biol. *95*, 325–332.
5. Darvin, P., Toor, S.M., Sasidharan Nair, V., and Elkord, E. (2018). Immune checkpoint inhibitors: Recent progress and potential biomarkers. Exp. Mol. Med. *50*, 1–11.
6. Mahoney, K.M., Freeman, G.J., and McDermott, D.F. (2015). The next immune-checkpoint inhibitors: PD-1/PD-L1 blockade in melanoma. Clin. Ther. *37*, 764–782.
7. Iwai, Y., Hamanishi, J., Chamoto, K., and Honjo, T. (2017). Cancer immunotherapies targeting the PD-1 signaling pathway. J. Biomed. Sci. *24*, 26.
8. Miliotou, A.N., and Papadopoulou, L.C. (2018). CAR T-cell therapy: A new era in cancer immunotherapy. Curr. Pharm. Biotechnol. *19*, 5–18.
9. Strati, P., and Neelapu, S.S. (2019). Chimeric antigen receptor-engineered T Cell therapy in lymphoma. Curr. Oncol. Rep. *21*, 38.
10. Davila, M.L., and Brentjens, R.J. (2016). CD19-targeted CAR T cells as novel cancer immunotherapy for relapsed or refractory B-cell acute lymphoblastic leukemia. Clin. Adv. Hematol. Oncol. *14*, 802–808.
11. Thorne, S.H., Liang, W., Sampath, P., Schmidt, T., Sikorski, R., Beilhack, A., and Contag, C.H. (2010). Targeting localized immune suppression within the tumor through repeat cycles of immune cell-oncolytic virus combination therapy. Mol. Ther. *18*, 1698–1705.
12. Rojas, J.J., and Thorne, S.H. (2012). Theranostic potential of oncolytic vaccinia virus. Theranostics *2*, 363–373.
13. Fenner, F., Henderson, D.A., Arita, I., Ježek, Z., and Ladnyi, I.D. (1988). Smallpox and Its Eradication (World Health Organization).
14. Kirn, D.H., and Thorne, S.H. (2009). Targeted and armed oncolytic poxviruses: a novel multi-mechanistic therapeutic class for cancer. Nat. Rev. Cancer *9*, 64–71.
15. Albarnaz, J.D., Torres, A.A., and Smith, G.L. (2018). Modulating vaccinia virus immunomodulators to improve immunological memory. Viruses *10*, 101.
16. Lawson, K.A., Sousa, C.M., Zhang, X., Kim, E., Akthar, R., Caumanns, J.J., Yao, Y., Mikolajewicz, N., Ross, C., Brown, K.R., et al. (2020). Functional genomic landscape of cancer-intrinsic evasion of killing by T cells. Nature *586*, 120–126.
17. Barr, A.M., Silva, A., Prato, S., Belz, G.T., Maraskovsky, E., and Baz Morelli, A. (2020). Therapeutic ISCOMATRIX™ adjuvant vaccine elicits effective anti-tumor immunity in the TRAMP-C1 mouse model of prostate cancer. Cancer Immunol. Immunother. *69*, 1959–1972.
18. Trumpfheller, C., Caskey, M., Nchinda, G., Longhi, M.P., Mizenina, O., Huang, Y., Schlesinger, S.J., Colonna, M., and Steinman, R.M. (2008). The microbial mimic poly IC induces durable and protective $CD4^+$ T cell immunity together with a dendritic cell targeted vaccine. Proc. Natl. Acad. Sci. USA *105*, 2574–2579.
19. Ammi, R., De Waele, J., Willemen, Y., Van Brussel, I., Schrijvers, D.M., Lion, E., and Smits, E.L.J. (2015). Poly(I:C) as cancer vaccine adjuvant: Knocking on the door of medical breakthroughs. Pharmacol. Ther. *146*, 120–131.
20. Zhu, X., Fallert-Junecko, B.A., Fujita, M., Ueda, R., Kohanbash, G., Kastenhuber, E.R., McDonald, H.A., Liu, Y., Kalinski, P., Reinhart, T.A., et al. (2010). Poly-ICLC promotes the infiltration of effector T cells into intracranial gliomas via induction of CXCL10 in IFN-α and IFN-γ dependent manners. Cancer Immunol. Immunother. *59*, 1401–1409.
21. Smith, G.L., Benfield, C.T.O., Maluquer de Motes, C., Mazzon, M., Ember, S.W.J., Ferguson, B.J., and Sumner, R.P. (2013). Vaccinia virus immune evasion: Mechanisms, virulence and immunogenicity. J. Gen. Virol. *94*, 2367–2392.
22. Sumner, R.P., Ren, H., and Smith, G.L. (2013). Deletion of immunomodulator C6 from vaccinia virus strain Western Reserve enhances virus immunogenicity and vaccine efficacy. J. Gen. Virol. *94*, 1121–1126.
23. García-Arriaza, J., Gómez, C.E., Sorzano, C.Ó., and Esteban, M. (2014). Deletion of the vaccinia virus N2L gene encoding an inhibitor of IRF3 improves the immunogenicity of modified vaccinia virus Ankara expressing HIV-1 antigens. J. Virol. *88*, 3392–3410.
24. Dai, P., Wang, W., Cao, H., Avogadri, F., Dai, L., Drexler, I., Joyce, J.A., Li, X.D., Chen, Z., Merghoub, T., et al. (2014). Modified vaccinia virus Ankara triggers type I IFN production in murine conventional dendritic cells via a cGAS/STING-mediated cytosolic DNA-sensing pathway. PLoS Pathog. *10*, e1003989.
25. Waibler, Z., Anzaghe, M., Ludwig, H., Akira, S., Weiss, S., Sutter, G., and Kalinke, U. (2007). Modified vaccinia virus Ankara induces Toll-like receptor-independent type I interferon responses. J. Virol. *81*, 12102–12110.
26. Van Hoecke, L., Riederer, S., Saelens, X., Sutter, G., and Rojas, J.J. (2020). Recombinant viruses delivering the necroptosis mediator MLKL induce a potent antitumor immunity in mice. OncoImmunology *9*, 1802968.
27. Drexler, I., Heller, K., Wahren, B., Erfle, V., and Sutter, G. (1998). Highly attenuated modified vaccinia virus Ankara replicates in baby hamster kidney cells, a potential host for virus propagation, but not in various human transformed and primary cells. J. Gen. Virol. *79*, 347–352.
28. Sutter, G., and Moss, B. (1992). Nonreplicating vaccinia vector efficiently expresses recombinant genes. Proc. Natl. Acad. Sci. USA *89*, 10847–10851.
29. Blanchard, T.J., Alcami, A., Andrea, P., and Smith, G.L. (1998). Modified vaccinia virus Ankara undergoes limited replication in human cells and lacks several immunomodulatory proteins: Implications for use as a human vaccine. J. Gen. Virol. *79*, 1159–1167.
30. Rojas, J.J., Sampath, P., Bonilla, B., Ashley, A., Hou, W., Byrd, D., and Thorne, S.H. (2016). Manipulating TLR signaling increases the anti-tumor T cell response induced by viral cancer therapies. Cell Rep. *15*, 264–273.
31. Unterholzner, L., Sumner, R.P., Baran, M., Ren, H., Mansur, D.S., Bourke, N.M., Randow, F., Smith, G.L., and Bowie, A.G. (2011). Vaccinia virus protein C6 is a virulence factor that binds TBK-1 adaptor proteins and inhibits activation of IRF3 and IRF7. PLoS Pathog. *7*, e1002247.
32. Smith, G.L. (2018). Vaccinia virus protein C6: A multifunctional interferon antagonist. Adv. Exp. Med. Biol. *1052*, 1–7.
33. Ferguson, B.J., Benfield, C.T.O., Ren, H., Lee, V.H., Frazer, G.L., Strnadova, P., Sumner, R.P., and Smith, G.L. (2013). Vaccinia virus protein N2 is a nuclear IRF3 inhibitor that promotes virulence. J. Gen. Virol. *94*, 2070–2081.
34. Peters, N.E., Ferguson, B.J., Mazzon, M., Fahy, A.S., Krysztofinska, E., Arribas-Bosacoma, R., Pearl, L.H., Ren, H., and Smith, G.L. (2013). A mechanism for the inhibition of DNA-PK-mediated DNA sensing by a virus. PLoS Pathog. *9*, e1003649.

35. Scutts, S.R., Ember, S.W., Ren, H., Ye, C., Lovejoy, C.A., Mazzon, M., Veyer, D.L., Sumner, R.P., and Smith, G.L. (2018). DNA-PK is targeted by multiple vaccinia virus proteins to inhibit DNA sensing. Cell Rep. *25*, 1953–1965.e4.

36. Gobillot, T.A., Humes, D., Sharma, A., Kikawa, C., and Overbaugh, J. (2020). The robust restriction of Zika virus by type-I interferon in A549 cells varies by viral lineage and is not determined by IFITM3. Viruses *12*, 503.

37. Jiao, P., Fan, W., Cao, Y., Zhang, H., Tian, L., Sun, L., Luo, T., Liu, W., and Li, J. (2020). Robust induction of interferon and interferon-stimulated gene expression by influenza B/Yamagata lineage virus infection of A549 cells. PLoS ONE *15*, e0231039.

38. Lehmann, M.H., Torres-Domínguez, L.E., Price, P.J., Brandmüller, C., Kirschning, C.J., and Sutter, G. (2016). CCL2 expression is mediated by type I IFN receptor and recruits NK and T cells to the lung during MVA infection. J. Leukoc. Biol. *99*, 1057–1064.

39. Volz, A., Jany, S., Freudenstein, A., Lantermann, M., Ludwig, H., and Sutter, G. (2018). E3L and F1L gene functions modulate the protective capacity of modified vaccinia virus Ankara immunization in murine model of human smallpox. Viruses *10*, 21.

40. Hanada, K.I., Yu, Z., Chappell, G.R., Park, A.S., and Restifo, N.P. (2019). An effective mouse model for adoptive cancer immunotherapy targeting neoantigens. JCI Insight *4*, e124405.

41. Kreiter, S., Vormehr, M., van de Roemer, N., Diken, M., Löwer, M., Diekmann, J., Boegel, S., Schrörs, B., Vascotto, F., Castle, J.C., et al. (2015). Mutant MHC class II epitopes drive therapeutic immune responses to cancer. Nature *520*, 692–696.

42. Shi, T., Song, X., Wang, Y., Liu, F., and Wei, J. (2020). Combining oncolytic viruses with cancer immunotherapy: Establishing a new generation of cancer treatment. Front. Immunol. *11*, 683.

43. Kano, Y., Iguchi, T., Matsui, H., Adachi, K., Sakoda, Y., Miyakawa, T., Doi, S., Hazama, S., Nagano, H., Ueyama, Y., and Tamada, K. (2016). Combined adjuvants of poly(I:C) plus LAG-3-Ig improve antitumor effects of tumor-specific T cells, preventing their exhaustion. Cancer Sci. *107*, 398–406.

44. Mikhaylova, I.N., Shubina, I.Z., Chkadua, G.Z., Petenko, N.N., Morozova, L.F., Burova, O.S., Beabelashvili, R.Sh., Parsunkova, K.A., Balatskaya, N.V., Chebanov, D.K., et al. (2018). Immunological monitoring for prediction of clinical response to antitumor vaccine therapy. Oncotarget *9*, 24381–24390.

45. Dai, P., Wang, W., Yang, N., Serna-Tamayo, C., Ricca, J.M., Zamarin, D., Shuman, S., Merghoub, T., Wolchok, J.D., and Deng, L. (2017). Intratumoral delivery of inactivated modified vaccinia virus Ankara (iMVA) induces systemic antitumor immunity via STING and Batf3-dependent dendritic cells. Sci. Immunol. *2*, eaal1713.

46. Fahy, A.S., Clark, R.H., Glyde, E.F., and Smith, G.L. (2008). Vaccinia virus protein C16 acts intracellularly to modulate the host response and promote virulence. J. Gen. Virol. *89*, 2377–2387.

47. Fedosyuk, S., Bezerra, G.A., Radakovics, K., Smith, T.K., Sammito, M., Bobik, N., Round, A., Ten Eyck, L.F., Djinović-Carugo, K., Usón, I., and Skern, T. (2016). Vaccinia virus immunomodulator A46: A lipid and protein-binding scaffold for sequestering host TIR-domain proteins. PLoS Pathog. *12*, e1006079.

48. Schröder, M., Baran, M., and Bowie, A.G. (2008). Viral targeting of DEAD box protein 3 reveals its role in TBK1/IKKε-mediated IRF activation. EMBO J. *27*, 2147–2157.

49. Alcamí, A., Symons, J.A., and Smith, G.L. (2000). The vaccinia virus soluble alpha/beta interferon (IFN) receptor binds to the cell surface and protects cells from the antiviral effects of IFN. J. Virol. *74*, 11230–11239.

50. Eaglesham, J.B., Pan, Y., Kupper, T.S., and Kranzusch, P.J. (2019). Viral and metazoan poxins are cGAMP-specific nucleases that restrict cGAS-STING signalling. Nature *566*, 259–263.

51. Livak, K.J., and Schmittgen, T.D. (2001). Analysis of relative gene expression data using real-time quantitative PCR and the $2^{-\Delta\Delta C(T)}$ method. Methods *25*, 402–408.

52. Wang, Y., Gong, D., Yao, C., Zheng, F., Zhou, T., Cao, Q., Zhu, X., Wang, M., and Zhu, J. (2020). Human monoclonal anti-TLR4 antibody negatively regulates lipopolysaccharide-induced inflammatory responses in mouse macrophages. Mol. Med. Rep. *22*, 4125–4134.

53. Mo, L., Zeng, Z., Deng, R., Li, Z., Sun, J., Hu, N., Shi, J., and Hu, Y. (2021). Hepatitis A virus-induced hsa-miR-146a-5p attenuates IFN-β signaling by targeting adaptor protein TRAF6. Arch. Virol. *166*, 789–799.

OMTO, Volume *22*

Supplemental information

Activation of interferon regulatory factor 3 by replication-competent vaccinia viruses improves antitumor efficacy mediated by T cell responses

Stephanie Riederer, Robert Fux, Michael H. Lehmann, Asisa Volz, Gerd Sutter, and Juan J. Rojas

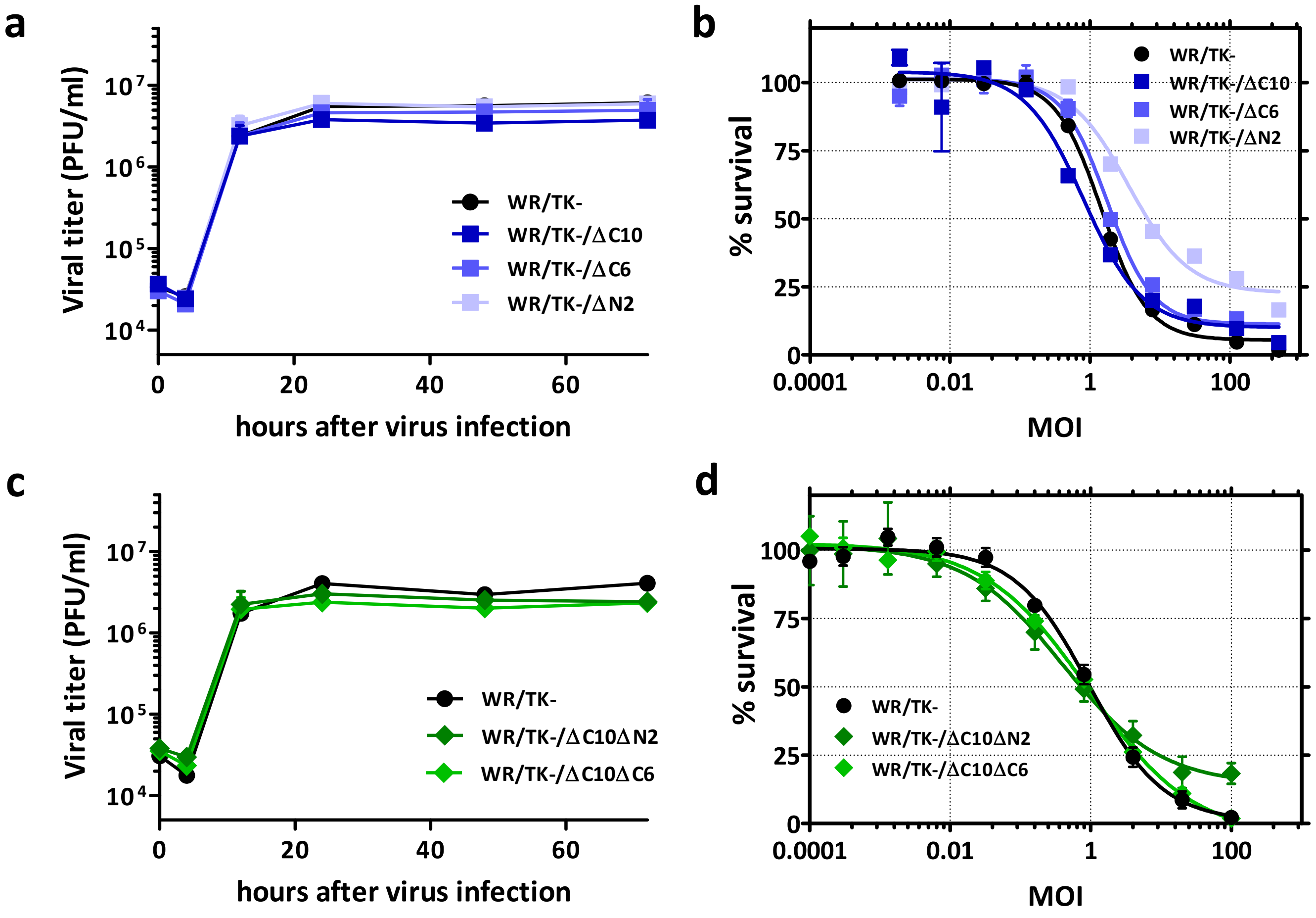

Supplementary Figure 1. Replication and cytotoxicity of various deletion mutant VACV. (a) Viral production of single-gene deleted viruses in HeLa cells. HeLa cells were infected with a multiplicity of infection (MOI) of 5 and, at indicated time points, samples were collected and viral titers were determined by plaque-assay. (b) Comparative cytotoxicity of single-gene deleted viruses in HeLa cells. HeLa cells were infected with indicated viruses at doses ranging from 200 to 0.001 PFU/cell. After 72 hours, % of cell death was evaluated. (c) Productive growth of double-gene deleted viruses in HeLa cells. HeLa cells were infected and treated as in (a) with indicated viruses. (d) Comparative cytotoxicity of double-gene deleted viruses in HeLa cells. HeLa cells were infected and treated as in (b) with indicated viruses.

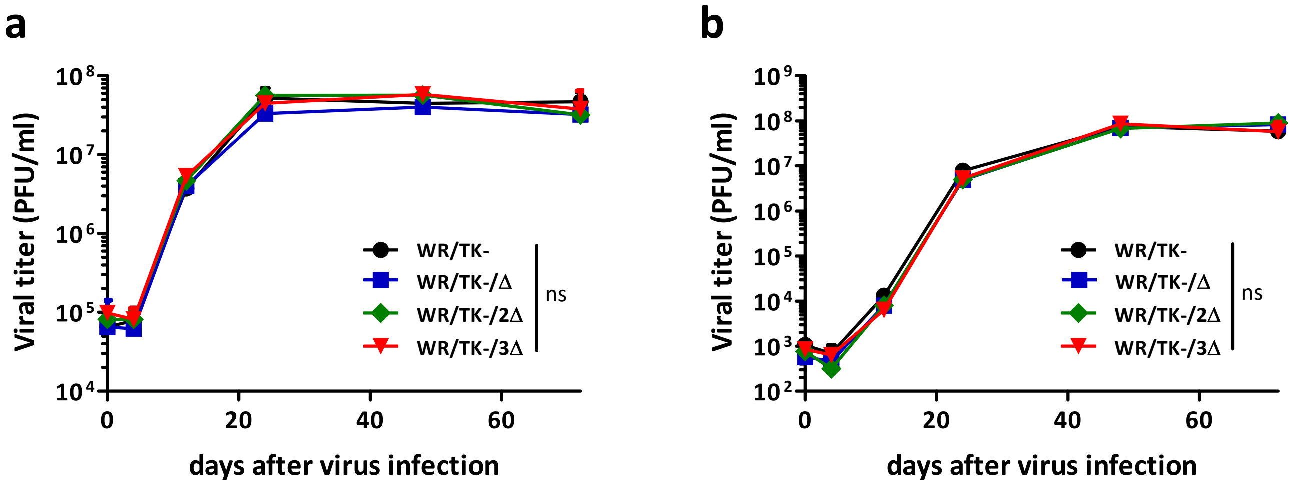

Supplementary Figure 2. Viral progeny production in A549 cells. A549 cells were infected with a multiplicity of infection (MOI) of 5 (a) or 0.05 (b) and, at indicated time points, samples were collected and viral titers were determined by plaque-assay. ns: not significant.

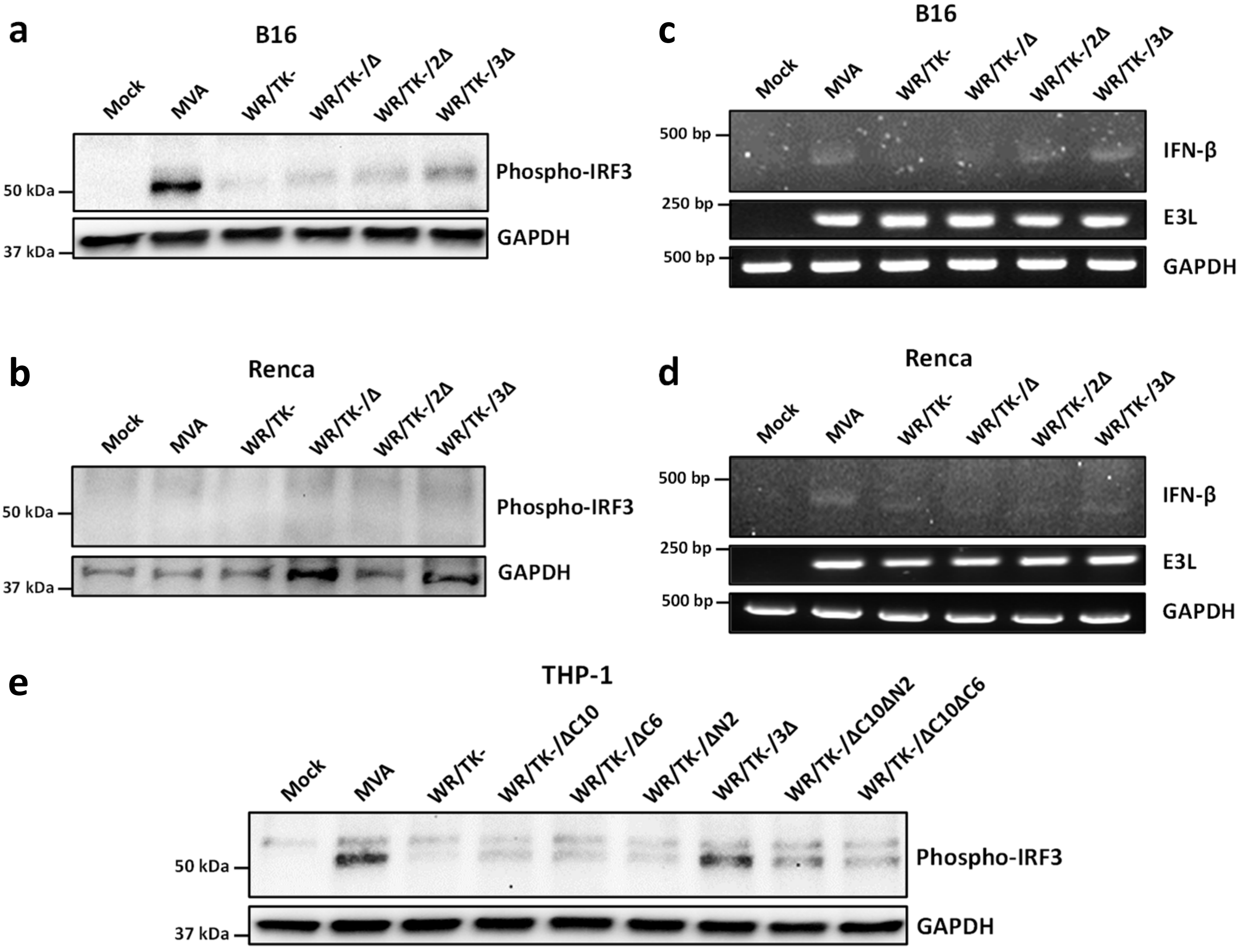

Supplementary Figure 3. Accumulation of genomic deletions is needed for activation of IRF3. (a-b) Phosphorylation of IRF3 after infection of mouse tumor cells. B16 (a) or Renca (b) cells were infected at a MOI of 10 and, 5 hours after infection, cells were lysed and Western Blot analysis was performed using a monoclonal antibody against phosphor-IRF3. MVA (Modified Vaccinia virus Ankara) served as a positive control and GAPDH-specific immunoblotting as a loading control. (c-d) Detection of IFN-β mRNA by RT-PCR after infection of mouse tumor cells. B16 (c) or Renca (d) cells were infected at a MOI of 5 and, 6 hours later, total RNA was obtained and the indicated mRNA of the indicated genes were amplified by RT-PCR. The VACV E3L mRNA was used as an infection control and GAPDH mRNA as a loading control. (e) Phosphorylation of IRF3 after infection with oncolytic viruses including all the possible combinations of target gene deletions. Human THP-1 cells were infected with indicated deletion mutant viruses at a MOI of 10 and, 5 hours after infection, cells were lysed and Western Blot analysis was performed using a monoclonal antibody against Phospho-IRF3. Vaccinia MVA strain was used as a positive control and GAPDH-blotting as a loading control.

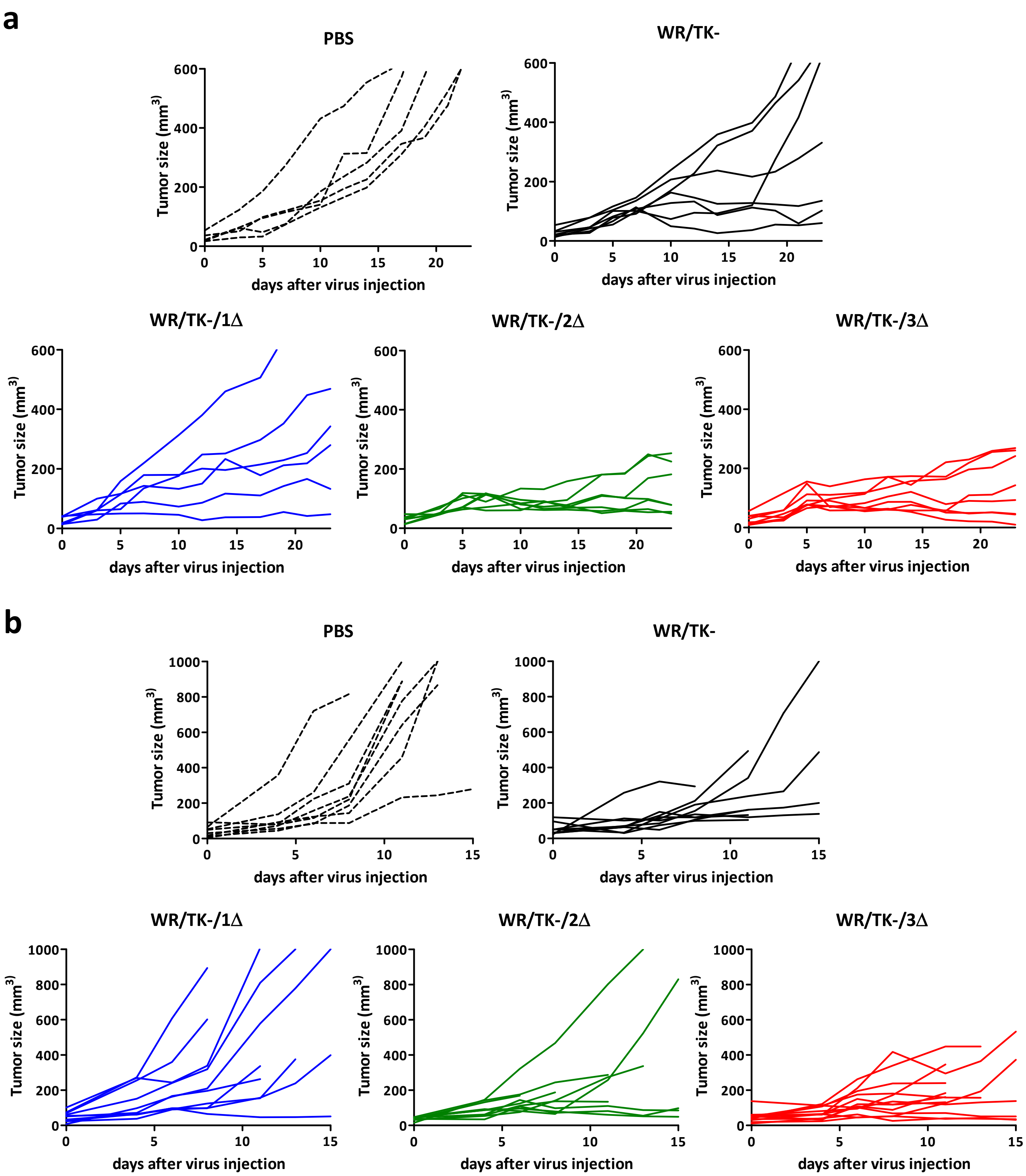

Supplementary Figure 4. Tumor growth curves of individual animals treated with candidate oncolytic VACV. Mean of treatments are shown in Figure 5. In short, mice with subcutaneous Renca (a) or B16 (b) tumors were treated by intratumoral injection with two doses of $1x10^7$ PFU of indicated viruses and tumor growth was monitored. Tumor growth of individual mice are depicted.
